# The 2026 ISCB Outstanding Service Award—Dr Philip E. Bourne

**DOI:** 10.1093/bioinformatics/btag280

**Published:** 2026-07-07

**Authors:** Mallory L Wiper

**Affiliations:** The International Society for Computational Biology, 525K East Market Street, RM330, Leesburg, VA 20176, United States


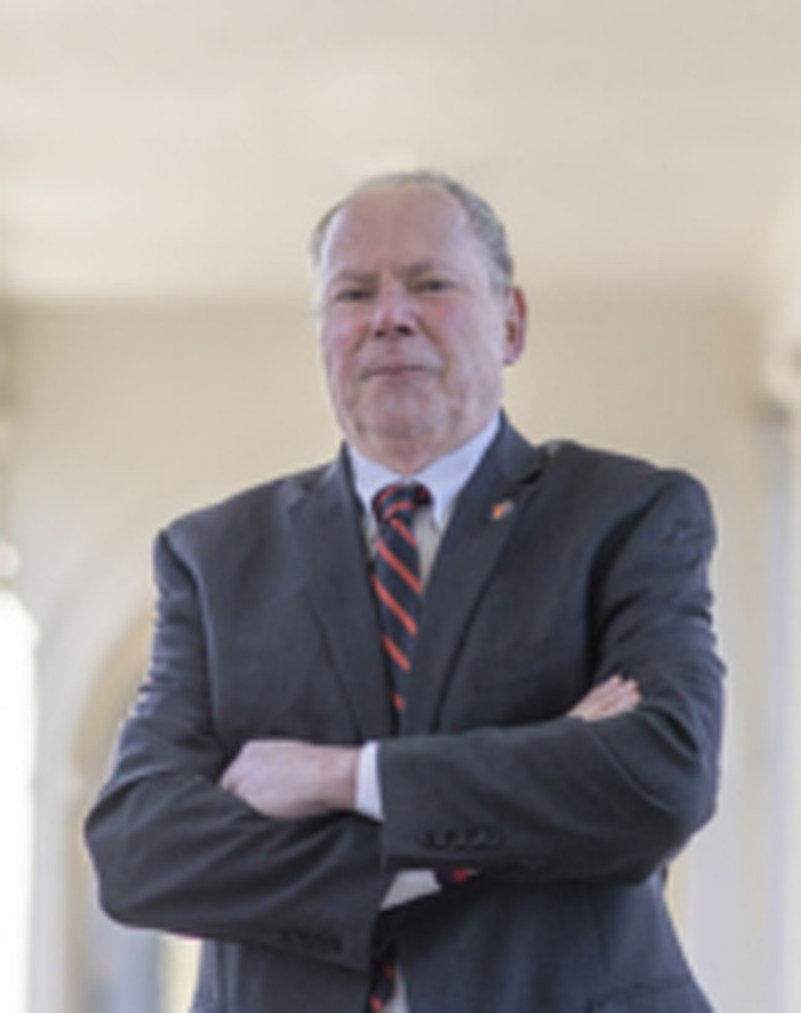


The annual Outstanding Service Award is presented to a member of the International Society for Computational Biology (ISCB) in recognition of their contributions to the betterment of computational biology through leadership, education, and service. This year, ISCB is proud to present this award to Dr Philip Bourne, whose work has advanced open, collaborative data science to further drive discovery in the field.

## 1 Early leadership in a growing field

Bourne first attended the Intelligent Systems for Molecular Biology (ISMB) conference in Cambridge in 1995—shortly before ISCB officially became a formal society—where attendance was about 300 people. A few years later, in 1999, he attended ISMB in Heidelberg, where attendance numbers had doubled. The next year, along with Mike Gribskov, Bourne co-chaired ISMB 2000, held at the University of California San Diego, where attendee numbers had doubled once again, now reaching over 1200. For Bourne, the formation of the society as a separate entity, coupled with the growth in attendee numbers at ISMB was clear evidence of how quickly the field of computational biology was expanding.

After co-chairing the ISMB meeting in 2000, Bourne became more formally involved with ISCB, taking up the mantle of ISCB President in 2002. During this time, Bourne worked with conference planner Stephanie Hagstrom to help streamline the organization of the meetings; he was also involved in bringing B.J. Morrison-McKay on as the first executive director of the Society.

## 2 Expanding the reach of computational biology

As ISCB president, Bourne strongly advocated for ISCB to be international in practice, not just in name. His commitment to global engagement took ISMB 2006 to Brazil, marking the Society’s early efforts to expand the conference beyond its traditional North American and European locations.

In addition to greater global reach, Bourne felt very strongly that ISCB needed a journal, one that was read not just by those in computational fields but by biologists. So, during his time as the chair of ISCB’s Publications Committee, he co-founded *PLOS Computational Biology* in 2005, the official journal of ISCB until 2019. This journal, he hoped, would give the field of computational biology broader reach and greater visibility.

Reflecting on the Society today, Bourne expressed his appreciation that ISCB has remained community-focused and that it “hasn’t outgrown its roots.” He’s happy to see that the Society continues to grow in membership and is particularly pleased with the continued role of the Student Council.

Looking ahead, Bourne believes ISCB should continue supporting students and fostering professional development within the computational biology community and emphasized the importance of ISCB maintaining its identity as a community-centered organization. To Bourne, the Society’s strength is in being a space for intellectual exchange, human connection, and international collaboration.

Drawing on his later career—including serving as the first Chief Data Officer at the National Institutes of Health (NIH) and founding the School of Data Science at the University of Virginia—Bourne encourages ISCB to remain attentive to broader developments in data science. In his view, understanding the evolving relationship between computational biology, biomedical data science, and the wider science ecosystem will be key to identifying future opportunities for the field.

## 3 Service to computational biology

One of Bourne’s most gratifying contributions to computational biology has been the creation of the “Ten Simple Rules” article series. This contribution stands out to him because of where the idea came from. At the 2005 Student Council Symposium in Detroit, Michigan, Bourne was asked to speak about what it takes to get research published. The unexpected energy and excitement of the symposium inspired Bourne to write “Ten Simple Rules for Getting Published.” The popularity of that article snowballed into the larger Ten Simple Rules article series, which has since become a widely read resource for teaching and professional development in computational biology and beyond.

Bourne also highlighted his time as the Chief Data Officer at the NIH as a significant role that allowed him to contribute to the computational biology community. In this national policy role, he advised members of the community on funding opportunities and on the finer points of data sharing and data sovereignty. After his time at NIH, Bourne founded the School of Data Science at the University of Virginia, where he focused on bridging developments in broader data science with biomedical data science to help communities like ISCB better understand emerging areas of importance and opportunity within the scientific landscape.

## 4 Advice for the next generation

When asked what advice he would give to students and early-career researchers about service opportunities, Bourne had a very clear message: Don’t laser-focus on research to the point of neglecting the community and get involved in service opportunities as early as possible.

Through opportunities like the ISCB Student Council, Bourne says, students can build networks of fellow academics and researchers who become peers and who may later become collaborators. He also emphasized that the human side of academia—the conferences, connections, and long-term professional relationships—is one of its most gratifying aspects. Overall, he encourages students to try to give as much time as they can to service to truly enrich their careers and the field.

## 5 Reflections on the Outstanding Service Award

Bourne expressed pride in the contributions he has made to the field of computational biology and in having played a role in ISCB’s early growth during its formative years. Though he admitted some surprise at being named the recipient of the Outstanding Service Award, he said he was honored by the recognition from the Society he has supported for many years.

## 6 A note from ISCB

Dr Philip E. Bourne, who passed away earlier this year, was a guiding force within computational biology and a steadfast advocate for the community and for ISCB, giving freely of his experience and expertise in service of strengthening the field.

ISCB is honored to celebrate Phil’s influence and lasting impact on computational biology and bioinformatics. We are also extraordinarily grateful to have had the opportunity to let Phil know he was this year’s winner of the Outstanding Contributions Award and to speak with him about his career and contributions before his passing. This article is a celebration of that conversation and of a life well lived in science.

